# MorphoTester: An Open Source Application for Morphological Topographic Analysis

**DOI:** 10.1371/journal.pone.0147649

**Published:** 2016-02-03

**Authors:** Julia M. Winchester

**Affiliations:** Interdepartmental Doctoral Program in Anthropological Sciences, Stony Brook University, Stony Brook, NY, United States of America; Monash University, AUSTRALIA

## Abstract

The increased prevalence and affordability of 3D scanning technology is beginning to have significant effects on the research questions and approaches available for studies of morphology. As the current trend of larger and more precise 3D datasets is unlikely to slow in the future, there is a need for efficient and capable tools for high-throughput quantitative analysis of biological shape. The promise and the challenge of implementing relatively automated methods for characterizing surface shape can be seen in the example of dental topographic analysis. Dental topographic analysis comprises a suite of techniques for quantifying tooth surfaces and component features. Topographic techniques have provided insight on mammalian molar form-function relationships and these methods could be applied to address other topics and questions. At the same time implementing multiple complementary topographic methods can have high time and labor costs, and comparability of data formats and approaches is difficult to predict. To address these challenges I present MorphoTester, an open source application for visualizing and quantifying topography from 3D triangulated polygon meshes. This application is Python-based and is free to use. MorphoTester implements three commonly used dental topographic metrics–Dirichlet normal energy, relief index, and orientation patch count rotated (OPCR). Previous OPCR algorithms have used raster-based grid data, which is not directly interchangeable with vector-based triangulated polygon meshes. A 3D-OPCR algorithm is provided here for quantifying complexity from polygon meshes. The efficacy of this metric is tested in a sample of mandibular second molars belonging to four species of cercopithecoid primates. Results suggest that 3D-OPCR is at least as effective for quantifying complexity as previous approaches, and may be more effective due to finer resolution of surface data considered here. MorphoTester represents an advancement in the automated quantification of morphology, and can be modified to adapt to future needs and priorities.

## Introduction

Improving techniques for quantifying morphological shape is a laudable goal with the potential to provide new insights on many questions pertaining to anatomical form. Recent advances in the speed and practicality of obtaining and analyzing three-dimensional (3D) data have led to a shift in quantitative morphological approaches (e.g. [1]). Techniques of geometric morphometrics have supplemented and to some degree supplanted traditional linear measurements of shape such as length and width [2,3]. MicroCT and laser scanning technology is becoming increasingly accessible, and this is allowing the creation of massive datasets of highly accurate 3D virtual replicas of anatomical elements belonging to extant and extinct species [4]. At the same time new scanning modalities are providing for the opportunity to non-destructively image structures that were previously inaccessible without destructive techniques. These include small scale structures such as enamel and dentine tubules, or deep anatomical features such as enamel-dentine interfaces for teeth or trabeculae for limb bones [5–8]. Quantity, quality, and diversity of 3D data are only likely to grow with time, and with this growth comes a need for high-throughput approaches to the analysis of morphological shape [9].

Topographic analytical methods that seek to quantify whole-surface morphology represent a potentially promising example of high-throughput shape analysis. These methods are commonly grouped using the term dental topographic analysis (DTA), reflecting a focus on quantitative assessment of the shape of tooth surfaces and component features [10–12]. This reflects how these techniques have been largely applied, though it is likely they could also be productively applied to non-dental structures. DTA was first implemented to characterize form-function correlations between mammalian molar topography and dietary behaviors [10,11,13–20]. These studies borrowed concepts from Geographic Information Systems (GIS) software to quantify teeth in a manner analogous to landscapes. It is possible that a better understanding of molar form-function correlations may provide insight into the dynamics of presumed adaptations of molar teeth for the mechanical breakdown of dietary food items. Quantitative topographic metrics such as average surface slope, relief, or angularity (the derivative of slope, or the change in slope across a surface) have been found to distinguish molar surfaces of folivorous and frugivorous primate species [13,14,16,17,20]. Specifically, folivorous species exhibit higher surface slope and relief reflecting higher cusps and crests, consistent with hypotheses of higher cusps and crests being an adaptation to overcome more mechanically resistant food items such as leaves.

Later implementations of DTA have quantified aspects of surface shape further removed from GIS concepts. Examples include the orientation patch count (OPC) metric, a method for measuring surface complexity [21,22], and the Dirichlet normal energy (DNE) metric, a method for quantifying surface bending using concepts from differential geometry [23]. Similar to the slope, relief, and angularity metrics described above, surface bending and complexity have been found to be effective for quantifying functionally relevant aspects of molar topography in extant and extinct mammalian species [6,12,21–30]. Analyses have also investigated correlations among shape descriptor metrics, and it has been shown that the use of multiple complementary shape descriptors increases statistical power for characterizing molar surface phenotypes [9,23, 31]. Quantification of molar topography using complementary descriptor metrics has been used to better characterize extant primate molar form in the context of dietary feeding behavior; to construct comparative datasets and models from extant species for the inference of paleodiet in extinct taxa; to reconstruct diet and ecomorphology in fossil species; and to assess relative contributions of the enamel-dentine junction and outer enamel surface to molar morphology [6,12,23,26,27,32,33].

Topographic analysis of morphology can be used to assess anatomical shape both broadly and deeply. Multiple metrics quantifying different axes of topographic form have been developed, and so topographic metrics used together in combination capture a multifaceted and complementary assessment of surface shape. Additionally, compared to many other techniques for the assessment of shape, dental topographic analyses rely less on specific landmarks or *a priori* subjective decisions. This “homology-free” nature allows the consideration of highly variable surfaces [20,21,23]. Using these methods it is possible to characterize morphology in diverse samples. In being relatively automated on the level of individual metrics compared to previous techniques, these methods can be considered high-throughput techniques for data acquisition.

In contrast to the advantages of this approach, topographic analyses have been implemented in ways that possibly limit their wider application. As the name suggests, dental topographic methods have been largely limited to the study of mammalian tooth form. While teeth make an excellent model system for the quantification of shape due to the robustness of enamel and the occlusal surface representing a definable surface domain, shape quantification is certainly not limited to the dentition. Given careful selection of desired surface regions, it is reasonable to think that a better understanding of many anatomical elements could be gained through comparative consideration of topographic bending, slope, relief, or complexity. One reason why topographic analyses have not been applied more widely is probably that much of the discussion of these methods has so far been limited to dental literature (but see [9]).

Practical factors also increase barriers to entry for use of these methods. Often published implementations of topographic metrics require expensive proprietary software. In fact, different proprietary applications are commonly used for each metric. As a result, monetary and labor costs of applying multiple topographic metrics are consequently high despite possible advantages. Metrics are also often performed on digital surface data formats that are not necessarily easily interchangeable (see below). This requires different approaches for the preparation of surface data, which itself increases costs and may also introduce unexpected variation into the surface data that topographic analyses seek to quantify. A single tool for performing topographic analyses on a uniform data format would help to minimize these challenges.

Here I present an application, MorphoTester, designed to address these challenges and to increase the ease of use and uniformity of topographic analyses. This application represents a software framework for visualizing and performing quantitative topographic analyses on 3D anatomical surface data. MorphoTester implements three common recent topographic metrics that quantify surface bending, relief, and complexity [20,21,23]. The application is free to use, and its source code and topographic metric algorithms (i.e., the steps taken by the program to calculate topographic metrics) are open source. This means that the program may be extended or modified to account for future needs. Taken together, MorphoTester implements high-throughput quantification of topography in a more detailed and uniform fashion than has been provided to date. In increasing the accessibility of topographic analyses, the aim of this software is to contribute to the goal of comprehensive relatively automated analysis of morphological variation.

## Materials and Methods

MorphoTester is an application framework for quantifying topographic shape from three-dimensional (3D) triangulated polygonal mesh data. It has been created using the Python programming language [34] and MorphoTester is free to use as well as open source. As a result, the topographic algorithms included and the base application code may be modified and reused under the terms of a GPL v2.0 (or later) license (see S1 Text for more details). Fundamentally MorphoTester represents a platform for inputting and visualizing polygonal mesh data and executing specific topographic algorithms on that data. Outputted results from topographic algorithms are quantitative descriptors of mesh shape [31]. In its default form this application is capable of calculating three topographic algorithms: Dirichlet normal energy (DNE, quantifies surface bending) [23], relief index (RFI, quantifies surface relief) [13,20], and orientation patch count rotated (OPCR, quantifies surface complexity) [21,22]. This framework is further extendable to include possible future topographic algorithms as well. Documentation for MorphoTester is provided as S1 Text, and source code and compiled executable files are available for download at http://morphotester.apotropa.com/.

### Accessing and using MorphoTester

For most users, compiled executable versions of MorphoTester are the easiest way to access the software. Compiled executables are provided for OSX and Windows operating systems. For OSX computers, this software is provided as a single file application bundle that can be run directly and placed in the Applications directory for continued access. MorphoTester for Windows is provided as a directory containing an executable file, *Morpho*.*exe*, and supporting data files. Users should run *Morpho*.*exe* to access the software. The program is operated entirely through the graphical user interface, and so no command line interaction is required. Executable versions provide the most direct path to using the program for “out of the box” topographic analysis. Users more familiar with Python can also run the application by interpreting the source code with Python installed. This first requires the installation of dependent Python packages (see below and S1 Text). If all dependencies have been met, the software can be opened by running the file *Morpho*.*py* as a script using the Python interpreter. Compiled and source-interpreted versions of MorphoTester have identical functionality. Compilation of source code for OSX and Windows was carried out using the Python packages *py2app* (www.pythonhosted.org/py2app) and *py2exe* (www.py2exe.org) respectively. Configuration files for executable compilation are available by request.

In addition to the website given above, MorphoTester source code and compiled executable releases are stored using Github (http://www.github.com/juliawinchester/morphotester/). Github is a major platform for storing and presenting code and code-related materials, and the linked repository is intended to serve as the long-term storage location for this software. The prominence of this platform ensures that this code is protected from the usual vagaries of university and personal webhosting. Additionally, Github has robust tools for communication and collaboration between users. The linked MorphoTester repository is equipped to serve as a central source of reports of software bugs and issues. It is also possible for users to “fork” or clone the software, establishing their own version for addressing problems or expanding features. Changes from software forks may then be merged back into the main repository. This provides interested users with direct access to the MorphoTester source code and easy tools for collaborative development of the software, and provides a direct path for continued maintenance.

When using MorphoTester, mesh data must be provided as Stanford Triangle or Polygon File Format (PLY), a common data format for triangulated polygonal meshes. Non-polygonal surface data such as point clouds can be readily triangulated to create polygon meshes using open source software such as Meshlab (http://meshlab.sourceforge.net/) or proprietary software such as Avizo (FEI Visualization Sciences Group) or Geomagic (3D Systems). PLY format data can also be easily converted to and from other common file formats such as.obj,.wrl, or.stl using free software such as Meshlab or meshconv [35]. MorphoTester is operated through a graphical user interface (S1 Fig). Users can load and visualize surface mesh files to be analyzed or select a directory for batch processing of analyses. Mesh files can be analyzed using any combination of DNE, RFI, and OPCR metrics by enabling or disabling these metrics prior to surface processing. For DNE and OPCR, submenus can be used to change parameters for analysis and enable visualizations of quantified topography on surface meshes. OPCR is visualized by coloring surface patches one of eight colors corresponding to patch orientation (see below). DNE is visualized using a color spectrum map across a surface mesh where warmer colors indicate greater surface bending at a polygon (e.g. Fig 3 from [23]). The DNE color map can be adjusted to show bending only relative to the current specimen or relative to an absolute range for comparing curvature between specimens. When processing individual specimens, results of topographic analyses are provided in a text console within the application. When batch processing a directory of specimens, a tab-separated values spreadsheet file listing results of topographic analyses is created in the specimen directory. This file can be opened using most spreadsheet software. Sample data for use with this software and reference topographic results can be downloaded from the above links. S1 Text contains more information on how to use this application and discusses analysis parameters in more detail.

### Program structure

Visualization and mathematical functions of MorphoTester are supported by pre-existing open source Python packages. These include the *Numpy* and *Scipy* stack [36], which provides data structures and functions for the large-format multidimensional arrays and matrices that are used to store polygonal mesh data. Mesh visualization is supported by the package *Mayavi* [37], and this package is integrated with *PyQt4* [38] and the non-Python open source library *Qt4* (www.qt.io) to implement the graphical user interface. *Matplotlib* [39] is used for data plotting tasks. A full list of package dependencies can be found in S1 Text. All of these backend packages have full documentation and so can be leveraged to modify MorphoTester code in a straightforward fashion for future needs.

In addition to being supported by third-party Python packages, this software incorporates and is supplied with Python packages and scripts to provide useful functions for working with triangulated polygon mesh data. Principal among these is *plython*, a package integrated with MorphoTester that provides functions for inputting, manipulating, and saving triangulated polygon mesh data within Python. Four other command line scripts are provided as well: *meshrotate*, which rotates individual PLY-format meshes in XYZ coordinate space; *meshrotate-batch*, which extends meshrotate to process multiple files; *PLYtoOFF*, which converts PLY-format mesh data to OFF-format; and *BINtoASC*, which converts PLY-format mesh from binary encoding to ASCII encoding. *BINtoASC* is useful as MorphoTester specifically interprets ASCII encoded PLY-format meshes, while some applications for modifying 3D meshes such as Geomagic only allow saving PLY-format meshes with binary encoding.

The code structure of MorphoTester is split between a single module containing support for visualization, the user interface, and integration of topographic shape metrics (*Morpho*.*py*), and three individual modules providing support for topographic shape metrics. Of the topographic algorithms, support for the calculation of DNE, RFI, and OPCR are provided by the modules *DNE*.*py*, *RFI*.*py*, and *OPC*.*py* respectively. Description of the calculation of these metrics follows.

### Dirichlet normal energy (DNE)

DNE can be briefly summarized as a quantification of the degree to which a surface mesh bends [23]. It is based on an application of a concept from differential geometry, Dirichlet’s energy, applied to the normal map of a mesh. Dirichlet’s energy is a measure of the variability of a function, and is termed energy because of applications to energy and action states in physics. DNE is also concerned with variability across a function, with that function being change in position across a three-dimensional surface. Surface variability includes both convexity and concavity, and as a result DNE increases with both types of shape change. In a continuous surface mesh case where surface polygons become arbitrarily small, the DNE method is equivalent to measuring the sum of squares of principal curvatures across a surface. This is in contrast to another recent morphological curvature measure, which averages polygon principal curvatures and correspondingly returns negative values for concavity and positive values for convexity [32,33].

**Fig 1:**
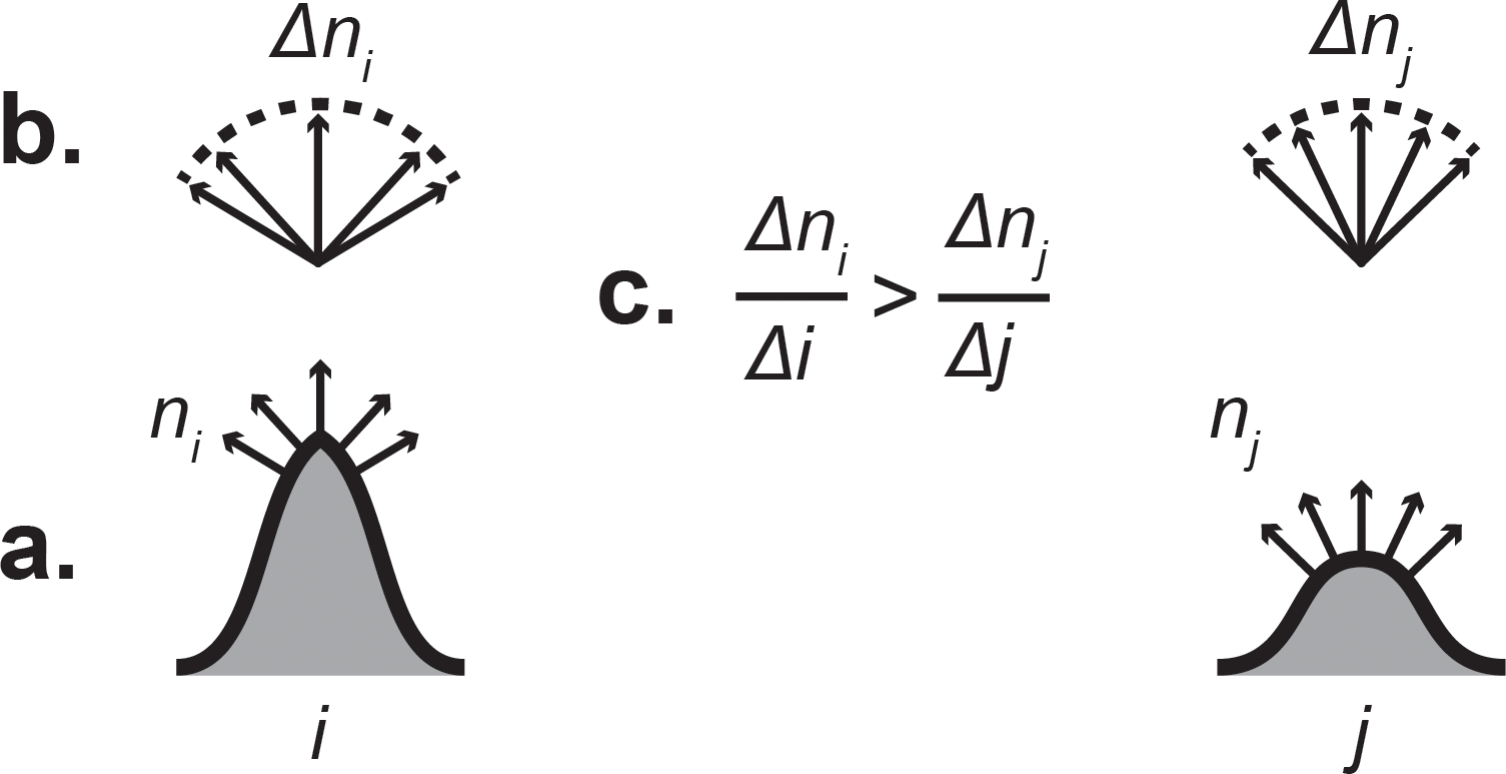
A simplistic two-dimensional diagram describing shape quantification using the Dirichlet normal energy method. (a) Given two surfaces *i* and *j*, normal vectors of magnitude one are derived for equal-length regions of interest. End-points of normal vectors define *n*_*i*_ and *n*_*j*_, the normal maps of *i* and *j* respectively. (b) *Δn* represents the change in position of end-points of normal vectors, or the change in the normal map. Superimposing origin points of normal vectors corrects *Δn* for the change in surface position (*Δi* or *Δj*). Arc length of superimposed normal vectors reflects degree of surface bending. (c) Stated explicitly, surface bending for a region of interest can be said to be characterized by change in the normal map (*Δn*) relative to change in surface position (*Δi* or *Δj*). Surface *i* shows greater bending. For the three-dimensional polygon mesh case used here, regions of interest are individual polygon vertices (see text for more details).

Bunn et al. [23] briefly described the mathematical background for DNE, but here I will expand on the method of the algorithm. DNE is calculated as the sum of energy values across a polygonal mesh surface. Energy values here equal the energy density of a polygon, *e(p)*, multiplied by polygonal face area. The energy density function *e(p)* quantifies change in the normal map around each polygon. While the explicit derivation of this function is given in detail below, it is possible to use a simplified two-dimensional diagram to understand how *e(p)* characterizes amount of bending from change across a surface normal map (Fig 1).

**Fig 2:**
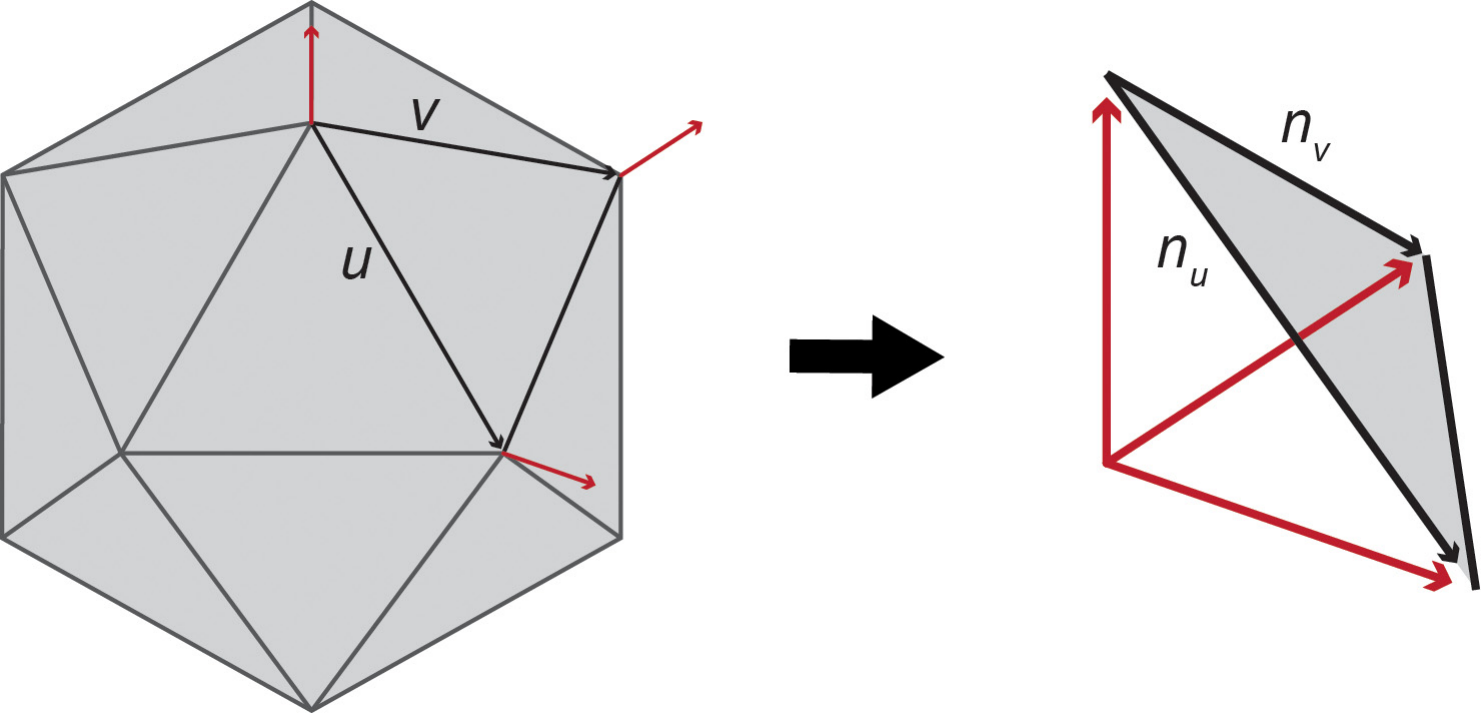
Diagram demonstrating edge vectors *u* and *v* of given polygon and approximated normal vectors (red) for polygon vertices. End-points of vertex normals form a polygon with edge vectors *n*_*u*_ and *n*_*v*_. Translating vertex normals to a common origin point visualizes spreading of *n*_*u*_ and *n*_*v*_ relative to spreading of *u* and *v*. Polygons on more curved surfaces will produce greater relative spreading of *n*_*u*_ and *n*_*v*_. *e(p)* quantifies relative spreading to calculate degree of surface bending per polygon.

To derive *e(p)*, normal vectors of unit length (i.e., having a magnitude of one) are first derived for each polygonal face comprising a mesh. Normal unit vectors for polygonal vertices are then approximated as the normalized average of normal vectors of triangle faces adjacent to each vertex. After producing approximated normal unit vectors for polygonal vertices, it is possible to consider two characterizations of polygon form. The first of these is defined by *u* and *v*, two vectors representing edges of a polygon (put another way, these are vectors representing change in surface position between vertices) (Fig 2). The second is defined by *n*_*u*_ and *n*_*v*_, which are derivatives of a surface normal map function *n* in the directions *u* and *v*. In a discrete surface mesh these are vectors representing edges of a polygon comprised of the endpoints of normal unit vectors derived from the original polygon (Fig 2).

Using *u* and *v*, it is possible to construct the matrix

$$G=\left(\begin{array}{cc} \left\langle u,u\right\rangle & \left\langle u,u\right\rangle \\ \left\langle u,v\right\rangle & \left\langle v,v\right\rangle \end{array}\right)$$

where ⟨∎,∎⟩ indicates the scalar Euclidean inner-product (dot product). These dot products characterize the magnitudes of *u* and *v* projected onto themselves and each other, and so *G* characterizes the spreading out of the original polygon. Similarly, using *n*_*u*_ and *n*_*v*_ it is possible to construct the matrix

$$H=\left(\begin{array}{cc} \left\langle n_{u},n_{u}\right\rangle & \left\langle n_{u},n_{v}\right\rangle \\ \left\langle n_{u},n_{v}\right\rangle & \left\langle n_{v},n_{v}\right\rangle \end{array}\right)$$

which characterizes the spreading out of the polygon normal map analogous to *G*. The bending of the surface around and at a polygon *p* is then derived as *e(p) = tr(G*^*-1*^*H)*. The trace of the product of matrices operates similarly to the dot product of vectors, and *e(p)* can simplistically be considered as the spreading of the polygonal normal map relative to the spreading of the original polygon.

Energy per polygon is *e(p)* multiplied by polygon area. Total DNE is then calculated from the sum of energy values of all polygons across a mesh surface, except for three conditional cases where polygonal energy may be discarded. (1) The DNE algorithm ignores energy from polygonal faces whose edges form part of the boundary of a hole in the mesh surface, as vertices related to these edges do not have a full complement of polygonal faces from which to approximate vertex normal vectors. For surfaces created from teeth, a single large inferior hole is often created through “cropping” of unnecessary surface, such as that inferior to the cervical margin [23]. (2) Optionally, energy values of polygons can be ignored where *G* produces a high condition number. Based on the ratio of the largest to smallest singular values in the singular value decomposition of a matrix, matrix condition numbers provide a measure of how close a matrix is to being singular. For *G* matrices with high condition numbers, very small changes in polygon vertex input are liable to produce large changes in energy output. Because of this, energy from these polygons is discarded. It is recommended that condition number checking be used when calculating DNE. (3) MorphoTester also allows optional discarding of energy or energy density values above a user specified outlier percentage. This can address surface meshes in which “noisy” polygons produce energy values out of proportion to the overall surface. Outlier removal of energy values above the 99.9^th^ percentile is enabled by default, but these settings can be easily user modified. Consistent outlier removal settings should be used for all specimens in a comparative sample as this setting does affect DNE results.

Higher and lower DNE values represent greater and lesser amounts respectively of bending across a surface. For the example of a primate molar tooth, higher and sharper cusps and crests as well as deeper and more acutely angled basins will produce higher DNE values. DNE is invariant to orientation or scaling of meshes, but quantified surface bending is proportional to the number of polygons comprising a mesh. This is because DNE is calculated as the sum of polygonal energy densities, and so meshes with greater numbers of polygons will necessarily exhibit higher DNE values than meshes of similar shape with fewer polygons. For analyzing samples of surface meshes, simplifying all meshes to a common number of polygons addresses this variance.

Previous analyses using DNE have employed Teether, a Matlab script for calculating DNE from polygonal surface data [12,23,26,27]. Teether has not been made widely available, and MorphoTester replicates the functionality of Teether completely with regards to DNE calculation and visualization. MorphoTester further corrects two errors in the Teether DNE algorithm and allows for more user control over analyses. Teether implements condition number checking as described above, but does not appropriately discard energy densities from polygons as a result. MorphoTester correctly implements condition number checking. Additionally, Teether forces meshes to be smoothed using an implicit fairing algorithm [40] prior to DNE calculation. If desired, MorphoTester can optionally perform an implicit fairing smooth for compatibility with Teether. MorphoTester also provides optional removal of energy or energy density values above a user specified percentile as outliers, as described above.

### Relief index (RFI)

For a mesh, RFI is defined as the ratio of three-dimensional surface area (*3da*) to two-dimensional surface area as projected on a plane parallel to the occlusal plane (*2da*) [13,20]. This metric has been calculated variously as $RFI=\frac{3da}{2da}\times 100$ [13,14,16,17] or as $RFI=ln\left(\frac{\sqrt{3da}}{\sqrt{2da}}\right)$ [12,20,23,27]. While measuring *3da* is straightforward, measuring *2da* from a concave 3D object is more algorithmically challenging. Some previous approaches calculating RFI from polygonal meshes have calculated *2da* by rotating meshes to maximize occlusal position, exporting a mesh view as a bitmap image, and then using image processing software to measure numbers of pixels in conjunction with a pixel-to-length scalebar [12,20]. MorphoTester automates this approach. After calculation, RFI is reported as a simple ratio of *3da* divided by *2da*. *3da* and *2da* are also provided so that RFI can be calculated using any desired formula.

### Orientation patch count rotated (OPCR)

Orientation patch count can be defined as the number of regions on a surface (“patches”) where adjacent polygons in a patch all face the same “compass” direction (i.e., have similarly angled normal vectors when projected on the XY plane). Most previous OPC analyses have sorted polygons into one of eight directional groups, each spanning a 45° arc, and so a perfect sphere should always have a count of eight orientation patches. OPC has been characterized as a surface complexity measure [21]. OPCR is a modification of the OPC approach designed to be more resistant to potential variation in specimen orientation on the XY plane [22]. OPCR addresses this by rotating individual molar specimens eight times across a total arc of 45° (5.625° per rotation), calculating OPC at each rotation. OPCR is the average of these eight variably rotated OPC values.

OPC has been applied to polygonal mesh surfaces [32,33,41], but OPCR has not. Previous implementations of OPCR have predominantly used the GIS software Surfer (Golden Software) and the application SurferManipulator [21]. SurferManipulator is designed to interact with Surfer for data preparation, and has stand-alone GIS functions for calculating OPCR [21]. This approach calculates OPCR from raster-based DEMs, which in this case are comprised of regularly-spaced columns and rows of data which correspond respectively with X and Y points. Each X and Y point pair is associated with at most one Z-axis elevation value, and so the DEMs represent a regularly spaced matrix of elevation information. This heightmap format differs from triangulated polygon meshes in a number of ways, principally in that DEMs cannot store two Z elevation values for one X-by-Y location (as in a sheer wall or an undercut) while polygonal meshes can [32]. This circumstance may not seem to be an issue for many kinds of anatomical specimens, including teeth, as biological surfaces rarely include perfectly vertical expanses. But a complex specimen exhibiting highly variable surface slope and significant change in height may give rise to surface regions that are intermittently vertical. A polygonal mesh may more accurately characterize a surface like this than a DEM. Additionally, the heightmap data model of the DEM format requires a more static Z-axis orientation compared to polygonal meshes. To increase comparability of metrics and to more accurately describe shape in complex surfaces, MorphoTester diverges from previous implementations of the OPCR metric by quantifying complexity from fully 3D triangulated polygon meshes instead of DEMs. This OPCR algorithm is introduced here. For clarity, I will refer to the triangulated mesh algorithm as 3D-OPCR and the method used by SurferManipulator as DEM-OPCR.

The 3D-OPCR algorithm requires only one parameter, a minimum patch size. This parameter indicates the minimum size in number of polygons for a patch to be counted toward an OPC value. To calculate OPC, the centroid of the surface mesh is translated to the origin of the XYZ coordinate system. Then normal unit vectors are derived for each polygonal face comprising the surface. Normal unit vectors are used to calculate the aspect of each face in the XY plane, and faces are sorted into one of eight groups by aspect. Each group represents an arc of 45 degrees. Contiguous polygons are then sorted into matching aspect groups, and iterative sorting of these arrays is used to construct a list of patches of contiguous polygons of identical aspect grouping. OPC is the number of patches at the minimum patch size or larger. To calculate OPCR this procedure is repeated eight times with the surface mesh being successively rotated 5.625° around the Z-axis, with the total mesh rotation being 45° by the eighth iteration. OPC values from each rotation are then averaged to give an OPCR value.

### Statistical analysis of 3D-OPCR

While the DNE and RFI algorithms used by MorphoTester implement analytical methods previously used in the literature, the 3D-OPCR algorithm has not been applied in prior studies. Because of this, 3D-OPCR as quantified by MorphoTester was compared to DEM-OPCR as measured by SurferManipulator using surface meshes created from plastic replica casts of second mandibular molar teeth of cercopithecoid primates (M_2_s, *n* = 36). This sample includes M_2_s belonging to 4 species: *Cercopithecus mitis* (*n = 10*), *Cercocebus atys* (*n* = 7), *Theropithecus gelada* (*n* = 9), and *Colobus guereza* (*n* = 10). M_2_s of these species are variable in shape and should make a suitable test case. Numerous previous dental topographic analyses have used M_2_s [12–14,16–20,23,26,27], and so this choice of tooth should increase the comparability of results. Only unworn or lightly worn specimens were chosen.

**Fig 3:**
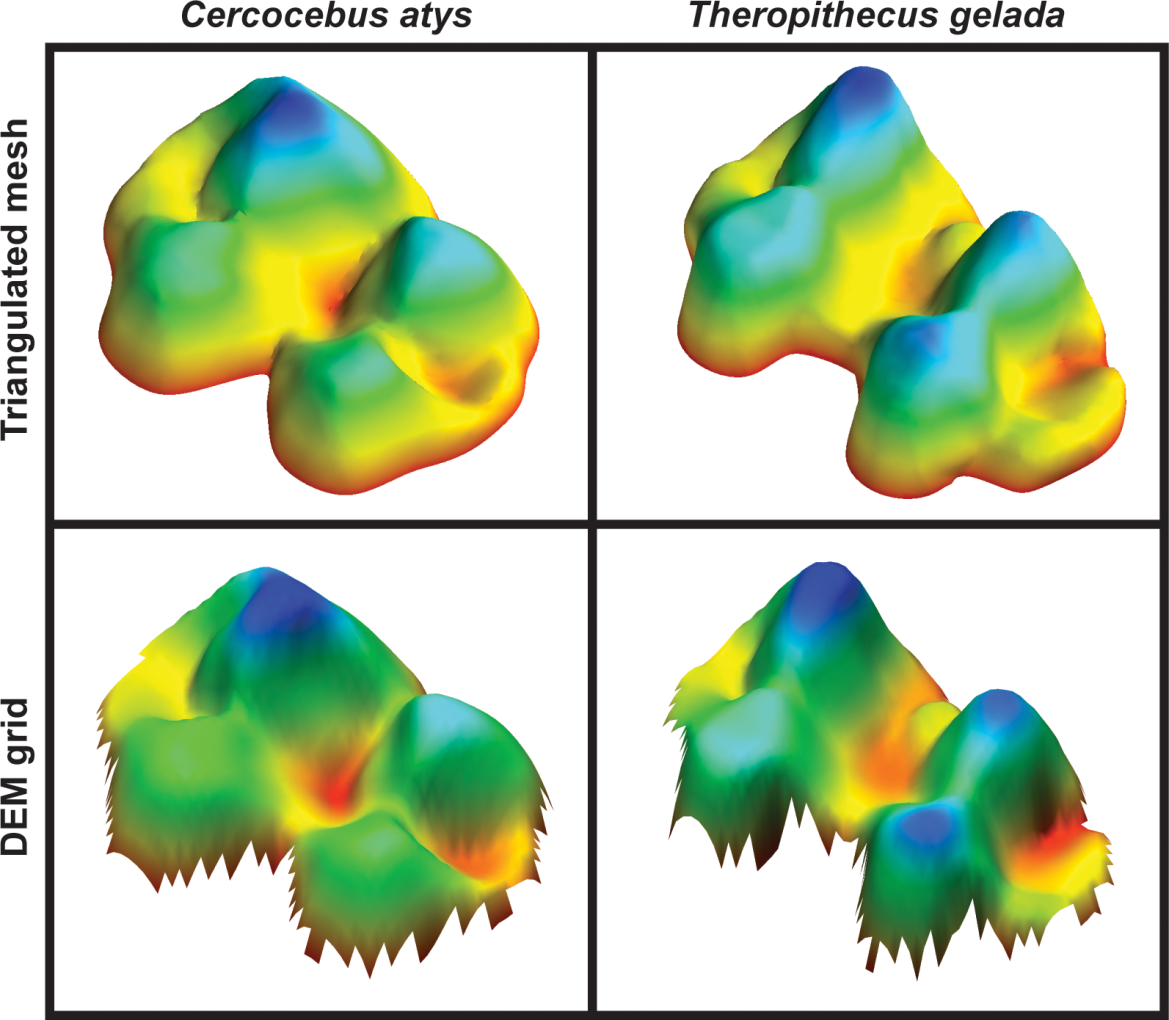
Comparison of triangulated mesh and DEM grid formats of second mandibular molar tooth surfaces for species *Cercocebus atys* and *Theropithecus gelada*. Teeth are presented in oblique perspective, with distal and buccal aspects toward bottom-right and bottom-left respectively. Color scaling reflects elevation. Triangulated mesh data is used for calculation of 3D-OPCR and DEM grid data is used for calculation of DEM-OPCR. Triangulated mesh data used here represents molar surface at a relatively finer resolution compared to DEM data.

To compare results from 3D-OPCR and DEM-OPCR algorithms, PLY-format surface meshes were first cropped to only include tooth surface above the lowest point on the central occlusal basin and then simplified and smoothed to remove noise. Meshes were simplified to 10,000 polygons and then smoothed across 100 iterations with a lambda parameter of 0.6 using the Simplifier and SmoothSurface modules of the Amira software (FEI Visualization Sciences Group). 3D-OPCR was then calculated using MorphoTester, with a minimum patch size of 5 polygons. To calculate DEM-OPCR, surface meshes were first converted to raster-based DEM format. This was done by first manually eliminating stacked elevation data by removing all polygonal faces not directly visible from a perspective parallel to the occlusal plane in Amira. After this, SurferManipulator’s file conversion tool was employed to convert data to Surfer DEM format. Original triangulated polygon surface meshes and resulting DEMs are presented as Fig 3. SurferManipulator was then used to calculate OPCR from converted DEMs, using previously documented methods [21–23]. DEMs were interpolated to include only 50 rows of data, which effectively normalizes tooth length per specimen. OPCR was then calculated using a minimum patch size of 3.

Minimum patch sizes for MorphoTester and SurferManipulator differ, and the larger minimum patch size for MorphoTester reflects the fact that triangulated polygon meshes as analyzed by MorphoTester encode more finely grained data resolution than the DEMs analyzed by SurferManipulator. While there is no exactly analogous measurement for comparison of resolution between polygon meshes and raster-based DEMs, it can be observed that most 10,000 face polygon meshes used in previous DTA studies contain over 5,000 data point XYZ vertices. Comparatively, the widest specimens in our sample approach 40 columns of XY data, and so after standardizing the number of rows to 50, the maximum number of Z-value elevation data points for a DEM would be 2,000. Nonetheless, minimum patch sizes are chosen largely arbitrarily.

After 3D-OPCR and DEM-OPCR were calculated, results were compared using SPSS v.17 (IBM). ANOVAs were run on each treatment using a species factor with *post hoc* pairwise comparison tests run using Tukey’s HSD. For all analyses, α = 0.05. F-values were compared between treatment ANOVAs as a measure of between-species variance relative to within-species variance, as were number of significant *post hoc* comparisons. It is predicted that ANOVAs of 3D-OPCR will exhibit higher F-values and more significant *post hoc* comparisons than ANOVAs of DEM-OPCR. Patterns of differences between 3D-OPCR and DEM-OPCR (ΔOPCR) were also investigated. Correlations between ΔOPCR and raw 3D-OCPR or DEM-OPCR were tested. An ANOVA was also run on ΔOPCR with a species factor with Tukey’s HSD *post hoc* pairwise comparison tests. It is predicted that ΔOPCR will not vary among species.

## Results

**Fig 4:**
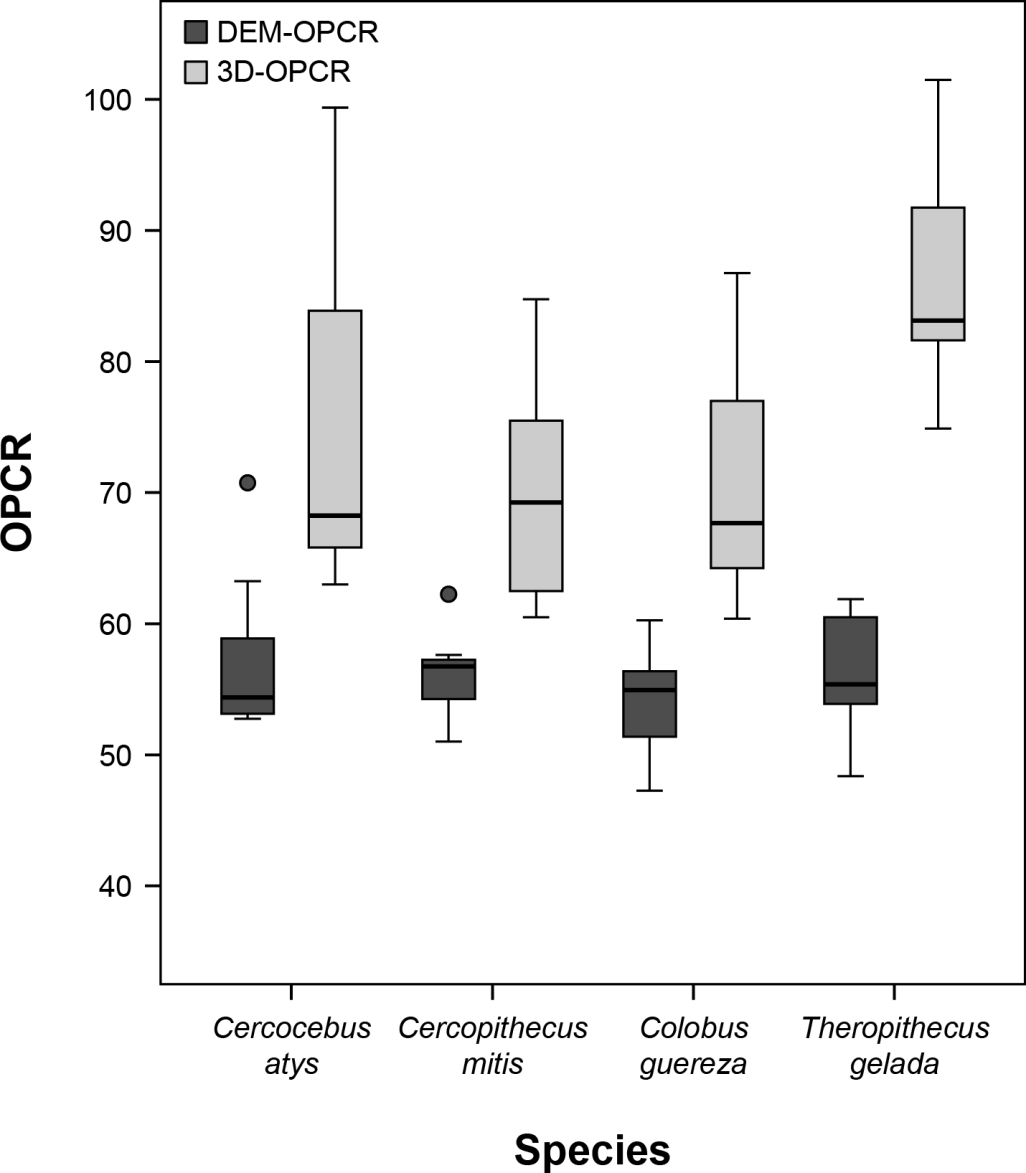
Box plot of DEM-OPCR and 3D-OPCR by species.

**Fig 5:**
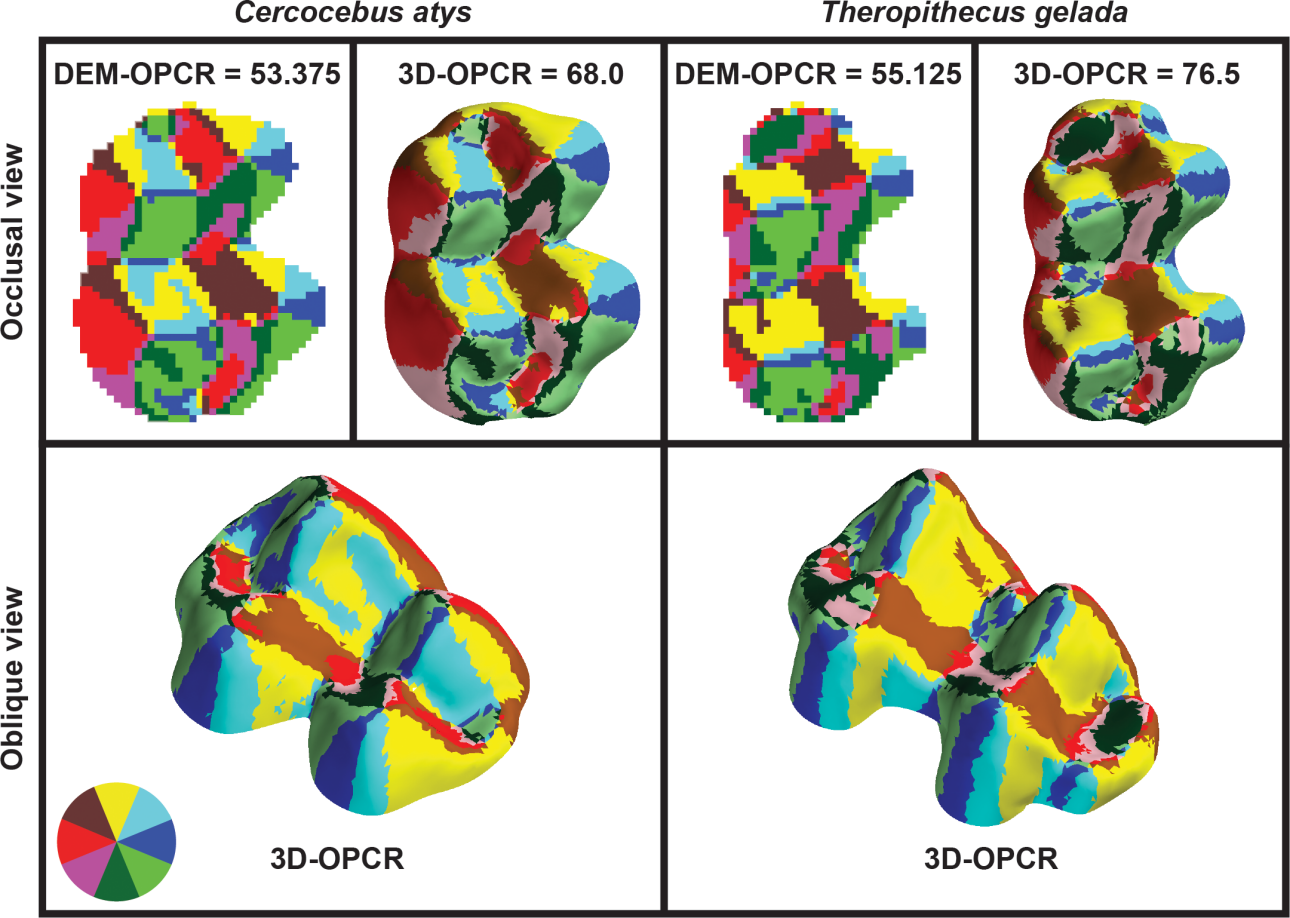
Results of DEM-OPCR and 3D-OPCR algorithms applied to molar tooth surfaces from Fig 3. Results from both algorithms are presented in occlusal perspective, with distal aspect at top and buccal aspect toward right. 3D-OPCR results are also shown in oblique perspective, with distal and buccal aspects toward bottom-right and bottom-left respectively. Color wheel at bottom left indicates patch aspect direction for occlusal perspective. 3D-OPCR results are presented with surface shading while DEM-OPCR results are not.

Descriptive statistics of 3D-OPCR, DEM-OPCR, and ΔOPCR by species are presented as Table 1, and represented as Fig 4. For sample M_2_s of *Cercocebus atys* and *T*. *gelada*, 3D-OPCR and DEM-OPCR are also graphically presented (Fig 5). Individual specimen data for these metrics are supplied as S1 Table, and OPC values at each rotation for 3D-OPCR and standard deviations of OPC per specimen are provided as S2 Table. Overall mean 3D-OPCR is higher than mean DEM-OPCR. Patterns of mean differences are similar between algorithm treatments. For both treatments, *Cercopithecus mitis* and *Colobus guereza* exhibit lower OPCR compared to *Cercocebus atys* and *T*. *gelada*, though for DEM-OPCR this difference is very small while for 3D-OPCR it is much larger. More variance within species can be observed for 3D-OPCR, but 3D-OPCR also evinces a clearer trend of difference between species than DEM-OPCR.

**Table 1:** Species mean by OPCR treatment.

		DEM-OPCR		3D-OPCR		ΔOPCR	
Species	*n*	mean	S.D.	mean	S.D	mean	S.D.
*Cercocebys atys*	7	57.41	6.932	75.71	13.798	18.30	9.133
*Cercopithecus mitis*	10	56.10	3.026	69.75	7.961	13.65	6.320
*Colobus guereza*	10	54.10	4.001	70.91	9.134	16.81	6.739
*Theropithecus gelada*	9	56.51	4.534	86.35	8.992	29.83	6.091
Total	36	55.90	4.571	75.38	11.601	19.48	9.182

ANOVAs were performed for each treatment using species factors, and the results of ANOVAs support observable trends for 3D-OPCR (Table 2). These analyses indicate 3D-OPCR significantly differs between species (*p* = 0.005), but that DEM-OPCR does not (*p* = 0.493). Correspondingly, ANOVA F-values are higher for the 3D-OPCR treatment, showing that the ratio of between-species variance to within-species variance is greater for 3D-OPCR relative to DEM-OPCR. *Post hoc* pairwise comparison tests using Tukey’s HSD were also performed. Due to the lack of significance for DEM-OPCR by ANOVA, only results from 3D-OPCR will be presented (Table 3). *T*. *gelada* was found to differ significantly in 3D-OPCR from both *Cercopithecus mitis* and *Colobus guereza*. *Cercocebus atys* was not found to significantly differ from any other species by 3D-OPCR.

**Table 2:** ANOVAs on OPCR treatments with species factor.

Treatment	*n*	MS b*	MS w*	*df*	*F*	*p*
DEM-OPCR	36	17.389	21.226	35	0.819	0.493
3D-OPCR	36	533.285	97.202	35	5.486	0.004

*MS b: mean square between species

MS w: mean square error within species

**Table 3:** Pairwise *post-hoc* comparisons of 3D-OPCR between species.

	*Cercopithecus mitis*	*Colobus guereza*	*Theropithecus gelada*
*Cercocebus atys*	5.964 (0.614)	4.802 (0.757)	10.633 (0.162)
*Cercopithecus mitis*		1.162 (0.993)	**16.597 (0.005)**
*Colobus guereza*			**15.435 (0.009)**

Cell values given as absolute mean differences between species, with Tukey’s HSD *p* following in parentheses. Bold indicates *p* < 0.05.

Significant positive relationships were found between ΔOPCR and both 3D-OPCR and DEM-OPCR (3D-OPCR: m = 0.736, b = -35.972, R^2^ = 0.864, p < 0.001; DEM-OPCR: m = 0.703, b = -19.806, R^2^ = 0.122; p = 0.036). ANOVA results indicate that ΔOPCR significantly varies by species, with *post hoc* pairwise comparison tests showing that ΔOPCR of *T*. *gelada* differs significantly from the three other species considered. No other species pairs differ in ΔOPCR (Tables 4 and 5).

**Table 4:** ANOVA on ΔOPCR with species factor.

*n*	MS b*	MS w*	*df*	*F*	*p*
36	461.819	48.921	35	9.440	<0.001

*MS b: mean square between species

MS w: mean square error within species.

**Table 5:** Pairwise *post-hoc* comparisons of ΔOPCR between species.

	*Cercopithecus mitis*	*Colobus guereza*	*Theropithecus gelada*
*Cercocebus atys*	4.654 (0.539)	1.491 (0.972)	**11.530 (0.013)**
*Cercopithecus mitis*		3.163 (0.744)	**16.183 (<0.001)**
*Colobus guereza*			**13.021 (0.002)**

Cell values given as absolute mean differences between species, with Tukey’s HSD *p* following in parentheses. Bold indicates *p* < 0.05.

## Discussion

Results from this study suggest that complexity as measured by the OPCR metric performed on triangulated polygon surface meshes (3D-OPCR) is at least as effective at partitioning differences in molar complexity as an OPCR metric performed on DEM data. 3D-OPCR is capable of significantly distinguishing between the species considered here, while DEM-OPCR is not. The lack of significance of DEM-OPCR in this case is interesting, given that DEM-OPCR has been shown to distinguish mammalian taxa with differing diets in other primate radiations including strepsirrhines and platyrrhines, as well as in carnivorans, rodents, and chiropterans [12,21,23,30]. It is probable that the significance by 3D-OPCR and lack of significance by DEM-OPCR is related to a difference in how *Theropithecus gelada* M_2_ complexity was characterized relative to other species. For 3D-OPCR *T*. *gelada* M_2_s were significantly more complex than M_2_s of *Cercopithecus mitis* or *Colobus guereza*, while DEM-OPCR was not found to significantly vary between species. Also while *T*. *gelada* was not found to significantly differ from *Cercocebus atys* in 3D-OPCR, mean 3D-OPCR does differ more between these species (10.64) than DEM-OPCR (-0.9). These results can be explained in several ways.

First, differences between treatments may reflect factors extrinsic to the molar specimens considered here. For relatively fine-resolution triangular polygon mesh data, the approach used here to convert polygonal meshes to DEM format may entail a loss of information which in this case reduces variation in complexity as quantified by DEM-OPCR. It is true that in converting data from a triangulated polygonal mesh to a DEM, some surface polygons are discarded. Probably more important is the fact that the DEM data was simplified to a much coarser resolution, having approximately one-fifth the number of surface data points compared to 3D-OPCR (see above and Fig 3). A reduction in variation of quantified complexity for the more simplified DEMs is supported by the relatively lower variance within and between species observed for DEM-OPCR. It must be noted that this high degree of DEM simplification is not necessary for calculating DEM-OPCR, and it is certainly possible to measure OPCR from DEMs with finer resolution. In fact, a recent analysis of dental complexity in fossil horses has shown that clearer evolutionary trends are apparent with increasingly fine DEM resolution [29]. At the same time a number of analyses of complexity of individual molars have employed the level of simplification used here [12,22,23,26,27,29], and so these results are relevant to a common method of applying DEM-OPCR.

It is also possible that the relatively low sample size considered here explains the lack of significance in DEM-OPCR. Previous analyses of strepsirrhine and platyrrhine primates had much greater overall sample sizes, although specimen numbers per taxon were generally similar to those considered here [12,23]. If trends from other primate radiations hold for cercopithecoids, then it is likely that a larger sample with more taxa with diverse molar morphology would show significant differences in DEM-OPCR. If this were the case, though, it is likely that a similar effect would be seen for 3D-OPCR and so a larger sample may not affect the relative performance of these metrics.

It is also possible that differences between treatments reflect factors intrinsic to the molar morphology of the species considered here. Namely, it is possible that magnitudes of differences between 3D-OPCR and DEM-OPCR are correlated with molar complexity or other shape aspects. This could explain significant differences between *T*. *gelada* and *Cercopithecus mitis* or *Colobus guereza* for 3D-OPCR where no similar differences were found for DEM-OPCR. Molars of *T*. *gelada* exhibit a morphology marked by complicated enamel infolding and rapid changes of slope compared to other species considered here. It is possible that loss of surface information and greater degrees of simplification associated with the DEM-OPCR approach used here affect quantified complexity for *T*. *gelada* to a greater degree than for other species. This is supported by the finding that differences between 3D-OPCR and DEM-OPCR are significantly greater in specimens with higher 3D-OPCR or DEM-OPCR values, and that *T*. *gelada* shows a greater difference between treatments than any other species considered here. If this does explain differences in patterns between 3D-OPCR and DEM-OPCR, observations here may be related to a recent finding that relief index values of molars of high-relief insectivorous strepsirrhine primates were more varied after being oriented to a common orientation by an automatic algorithm relative to lower-relief molars of other species [42]. That is, surface data with high crowns or otherwise significant vertical area (i.e., having great change in the Z axis) may be more sensitive to modifications of surface data related to the Z axis. Removing stacked elevation data in the Z axis is more likely to affect surfaces with more complex vertical area, and equal modifications of occlusal plane orientation in high and low crowned teeth may affect relief more in high-crowned teeth. Taken together these results suggests that topographic analysis is a powerful tool for quantitatively describing anatomical shape, but at the same time serious consideration of methodology is necessary to characterize results. Choice of pathway from specimen to quantified data is likely to affect observations in ways that are non-trivial and sometimes difficult to predict.

Topographic analyses of morphology have shown great promise for providing new ways to quantify complex aspects of surface shape. They are especially likely to be useful in contexts where functional adaptations may be more strongly linked to overall “emergent” geometry than to specific features comprising that geometry. As molar masticatory efficiency is likely more related to overall shearing, puncturing, or crushing potential than to the presence or absence of any particular cusp, molar form probably represents a good example of this [41]. And yet, teeth are unlikely to be the only suitable subject. If multiple distinct morphological configurations can be adapted to address functional challenges, then holistic homology-free shape descriptors are likely to be an effective quantitative tool for better understanding anatomical form-function relationships broadly. The software presented here provides a direct and unified approach to perform complementary topographic analyses on a uniform polygonal mesh data format. This tool also expands on the capabilities of previous implementations of topographic methods. Surface bending (DNE) can be quantified from surfaces with finer resolution (more polygon faces) than is possible for the Teether Matlab tool used by Bunn et al. [23]. MorphoTester provides a method for measuring surface complexity (OPCR) from triangulated polygon meshes that is at least as effective as approaches using DEM surface data. And this software includes a novel fully automated approach to measuring surface relief through the relief index metric.

As affordability and accessibility of scanning technologies increase over time, morphologists will continue to have access to progressively larger datasets of highly accurate 3D surfaces representing anatomical elements. To make sense of progressively expanding assemblages of 3D morphological data and to most efficiently derive scientific insights from this data, it is going to be necessary to have high-throughput analytical tools designed to work with large datasets to extract as much information as possible. These techniques are currently being developed, but in some cases their wider application is hampered by high labor and financial costs associated with proprietary software and a diversity of methodological pathways from data to results. The free open source software presented here allows more automated and comprehensive implementation of morphological analytic methods. This application has been designed to quantify detailed descriptors of complementary aspects of shape from complicated anatomical surfaces and to do so across large datasets including diverse anatomical forms. In this context, this software is an evolutionary step toward tools for deeper and broader considerations of morphological variation.

## Supporting Information

S1 Fig MorphoTester user interface. (TIF)

S1 Table DEM-OPCR and 3D-OPCR values for individual specimens. (DOCX)

S2 Table OPC values at each rotation per specimen for 3D-OPCR. (DOCX)

S1 Text MorphoTester documentation. (DOCX)
