# Supplementary figures and images for: MorphoTester: An Open Source Application for Morphological Topographic Analysis
Source: PLoS One. 2016 Feb 3;11(2):e0147649. doi: 10.1371/journal.pone.0147649 (PMC4739702; doi:10.1371/journal.pone.0147649)

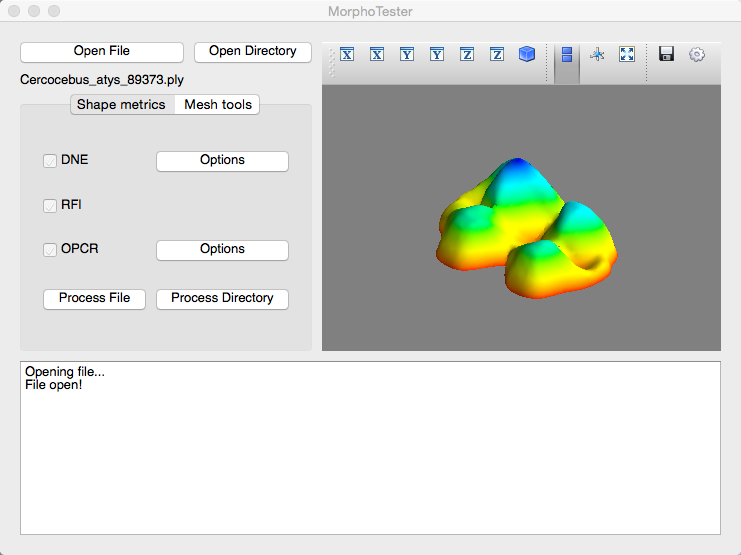

Supplement: S1 Fig — (TIF) [file pone.0147649.s001.tif]
