# Supplementary material for: MorphoTester: An Open Source Application for Morphological Topographic Analysis
Source: PLoS One. 2016 Feb 3;11(2):e0147649. doi: 10.1371/journal.pone.0147649 (PMC4739702; doi:10.1371/journal.pone.0147649)
Supplement: S1 Table — (DOCX) [file pone.0147649.s002.docx]

S1 Table. DEM-OPCR and 3D-OPCR values for individual specimens.

| Species | Museum | Specimen | DEM-OPCR | 3D-OPCR | | ΔOPCR |
| --- | --- | --- | --- | --- | --- | --- |
| *Cercocebus atys* | AMNH | 70063 | 52.75 | 68.25 | 15.5 | |
| *Cercocebus atys* | AMNH | 70385 | 54.5 | 63.625 | 9.125 | |
| *Cercocebus atys* | AMNH | 77777 | 52.875 | 63 | 10.125 | |
| *Cercocebus atys* | AMNH | 89373 | 53.375 | 68 | 14.625 | |
| *Cercocebus atys* | MNHN | 1982-1065 | 54.375 | 87.5 | 33.125 | |
| *Cercocebus atys* | MNHN | 1962-1437 | 63.25 | 80.25 | 17 | |
| *Cercocebus atys* | MNHN | 1962-1431 | 70.75 | 99.375 | 28.625 | |
| *Cercopithecus mitis* | AMNH | 52354 | 57.25 | 60.5 | 3.25 | |
| *Cercopithecus mitis* | AMNH | 52355 | 57.625 | 75.5 | 17.875 | |
| *Cercopithecus mitis* | AMNH | 52364 | 56.75 | 64 | 7.25 | |
| *Cercopithecus mitis* | NMNH | 236996 | 62.25 | 84.75 | 22.5 | |
| *Cercopithecus mitis* | NMNH | 259446 | 53.375 | 66.75 | 13.375 | |
| *Cercopithecus mitis* | NMNH | 452544 | 54.25 | 71.75 | 17.5 | |
| *Cercopithecus mitis* | NMNH | 452547 | 54.625 | 62.125 | 7.5 | |
| *Cercopithecus mitis* | NMNH | 452548 | 56.75 | 77.875 | 21.125 | |
| *Cercopithecus mitis* | NMNH | 452552 | 51 | 62.5 | 11.5 | |
| *Cercopithecus mitis* | NMNH | 452554 | 57.125 | 71.75 | 14.625 | |
| *Colobus guereza* | AMNH | 52236 | 51.375 | 64.25 | 12.875 | |
| *Colobus guereza* | BMNH | 28.11.11.2 | 55.375 | 68.25 | 12.875 | |
| *Colobus guereza* | BMNH | 14.1.24.1 | 57.875 | 85.25 | 27.375 | |
| *Colobus guereza* | BMNH | 72.152 | 60.25 | 77 | 16.75 | |
| *Colobus guereza* | BMNH | 40.8 | 54.5 | 70.625 | 16.125 | |
| *Colobus guereza* | BMNH | 1.4.6.1 | 56.125 | 86.75 | 30.625 | |
| *Colobus guereza* | BMNH | 54.762 | 56.375 | 66.25 | 9.875 | |
| *Colobus guereza* | BMNH | 24.8.6.4 | 49.125 | 63.25 | 14.125 | |
| *Colobus guereza* | BMNH | 1938.9.9.4 | 52.75 | 67.125 | 14.375 | |
| *Colobus guereza* | MNHN | 163627 | 47.25 | 60.375 | 13.125 | |
| *Theropithecus gelada* | MNHN | 1971-10 | 48.375 | 82.125 | 33.75 | |
| *Theropithecus gelada* | MNHN | 1934-1419 | 55.375 | 83.125 | 27.75 | |
| *Theropithecus gelada* | MNHN | A-1.440 | 60.5 | 89.25 | 28.75 | |
| *Theropithecus gelada* | MNHN | 1962-1467 | 61.875 | 96.375 | 34.5 | |
| *Theropithecus gelada* | MNHN | 1972-360 | 59.5 | 91.75 | 32.25 | |
| *Theropithecus gelada* | MNHN | 1969-451 | 61.125 | 101.5 | 40.375 | |
| *Theropithecus gelada* | MNHN | 1963-58 | 55.125 | 76.5 | 21.375 | |
| *Theropithecus gelada* | MNHN | 1931-836 | 53.875 | 81.625 | 27.75 | |
| *Theropithecus gelada* | NMNH | 305107 | 52.875 | 74.875 | 22 | |

* Museum attributions: AMNH - American Museum of Natural History, New York City; BMNH - Natural History Museum, London; MNHN - Muséum national d’Histoire naturelle, Paris; NMNH, National Museum of Natural History, Washington. D.C. Δ: Change from DEM-OPCR to 3D-OPCR.
