# Supplementary material for: MorphoTester: An Open Source Application for Morphological Topographic Analysis
Source: PLoS One. 2016 Feb 3;11(2):e0147649. doi: 10.1371/journal.pone.0147649 (PMC4739702; doi:10.1371/journal.pone.0147649)
Supplement: S2 Table — (DOCX) [file pone.0147649.s003.docx]

S2 Table. OPC at each rotation for 3D-OPCR. 3D-OPCR is average of all OPC values per specimen.

|  |  | OPC at rotation: | | | | | | | |  |
| --- | --- | --- | --- | --- | --- | --- | --- | --- | --- | --- |
| Species | Specimen | 1 | 2 | 3 | 4 | 5 | 6 | 7 | 8 | Std. Dev. |
| *Cercocebus atys* | 1065 | 82 | 94 | 91 | 93 | 86 | 84 | 84 | 86 | 4.536 |
| *Cercocebus atys* | 1437 | 81 | 86 | 81 | 85 | 78 | 83 | 78 | 78 | 3.196 |
| *Cercocebus atys* | 1431 | 98 | 96 | 97 | 95 | 105 | 97 | 105 | 103 | 4.140 |
| *Cercocebus atys* | 70063 | 71 | 68 | 70 | 72 | 71 | 68 | 63 | 69 | 2.828 |
| *Cercocebus atys* | 70385 | 65 | 64 | 60 | 61 | 66 | 65 | 62 | 66 | 2.326 |
| *Cercocebus atys* | 77777 | 58 | 58 | 65 | 64 | 70 | 64 | 66 | 59 | 4.309 |
| *Cercocebus atys* | 89373 | 70 | 68 | 68 | 63 | 67 | 70 | 68 | 70 | 2.330 |
| *Cercopithecus mitis* | 236996 | 83 | 80 | 85 | 87 | 89 | 84 | 85 | 85 | 2.659 |
| *Cercopithecus mitis* | 259446 | 60 | 71 | 75 | 72 | 68 | 63 | 62 | 61 | 5.732 |
| *Cercopithecus mitis* | 452544 | 71 | 75 | 75 | 74 | 73 | 68 | 68 | 70 | 2.915 |
| *Cercopithecus mitis* | 452547 | 64 | 64 | 61 | 59 | 59 | 62 | 66 | 62 | 2.475 |
| *Cercopithecus mitis* | 452548 | 78 | 81 | 84 | 76 | 72 | 77 | 75 | 80 | 3.758 |
| *Cercopithecus mitis* | 452552 | 62 | 61 | 62 | 61 | 64 | 65 | 63 | 62 | 1.414 |
| *Cercopithecus mitis* | 452554 | 72 | 68 | 67 | 74 | 73 | 72 | 74 | 74 | 2.765 |
| *Cercopithecus mitis* | 52354 | 60 | 62 | 62 | 59 | 63 | 60 | 57 | 61 | 1.927 |
| *Cercopithecus mitis* | 52355 | 83 | 83 | 76 | 81 | 76 | 76 | 80 | 82 | 3.159 |
| *Cercopithecus mitis* | 52364 | 60 | 62 | 60 | 67 | 69 | 67 | 60 | 67 | 3.854 |
| *Colobus guereza* | 52236 | 68 | 60 | 64 | 62 | 59 | 64 | 65 | 72 | 4.234 |
| *Colobus guereza* | 11112 | 67 | 69 | 68 | 68 | 65 | 65 | 72 | 72 | 2.712 |
| *Colobus guereza* | 1241 | 83 | 86 | 89 | 83 | 82 | 82 | 85 | 92 | 3.615 |
| *Colobus guereza* | 152 | 84 | 77 | 84 | 77 | 72 | 73 | 74 | 75 | 4.660 |
| *Colobus guereza* | 163627 | 60 | 58 | 66 | 59 | 61 | 59 | 61 | 59 | 2.504 |
| *Colobus guereza* | 408 | 71 | 72 | 75 | 78 | 70 | 66 | 66 | 67 | 4.340 |
| *Colobus guereza* | 461 | 93 | 89 | 96 | 88 | 86 | 80 | 82 | 80 | 5.922 |
| *Colobus guereza* | 762 | 70 | 67 | 69 | 64 | 64 | 64 | 65 | 67 | 2.375 |
| *Colobus guereza* | 864 | 62 | 69 | 66 | 67 | 62 | 59 | 63 | 48 | 6.503 |
| *Colobus guereza* | 994 | 71 | 67 | 65 | 66 | 63 | 69 | 68 | 68 | 2.475 |
| *Theropithecus gelada* | 10 | 79 | 76 | 82 | 78 | 86 | 88 | 85 | 83 | 4.190 |
| *Theropithecus gelada* | 1419 | 81 | 87 | 79 | 86 | 89 | 83 | 79 | 81 | 3.796 |
| *Theropithecus gelada* | 1440 | 85 | 89 | 96 | 88 | 89 | 94 | 88 | 85 | 3.919 |
| *Theropithecus gelada* | 1467 | 101 | 89 | 97 | 100 | 95 | 94 | 91 | 104 | 5.125 |
| *Theropithecus gelada* | 305107 | 70 | 75 | 76 | 73 | 79 | 75 | 73 | 78 | 2.900 |
| *Theropithecus gelada* | 360 | 90 | 93 | 92 | 100 | 92 | 91 | 87 | 89 | 3.845 |
| *Theropithecus gelada* | 451 | 103 | 108 | 104 | 103 | 100 | 101 | 96 | 97 | 3.891 |
| *Theropithecus gelada* | 58 | 79 | 80 | 77 | 72 | 75 | 75 | 79 | 75 | 2.726 |
| *Theropithecus gelada* | 836 | 79 | 78 | 84 | 87 | 81 | 82 | 80 | 82 | 2.875 |
