# Supplementary material for: MorphoTester: An Open Source Application for Morphological Topographic Analysis
Source: PLoS One. 2016 Feb 3;11(2):e0147649. doi: 10.1371/journal.pone.0147649 (PMC4739702; doi:10.1371/journal.pone.0147649)
Supplement: S1 Text — (DOCX) [file pone.0147649.s004.docx]

**MorphoTester Documentation**

*The most recent updated version of this document can be found in the MorphoTester Github repository (http://www.github.com/juliawinchester/morphotester/) as a README.txt file.*

MorphoTester is a scientific computing application for quantifying topographic shape from three-dimensional triangulated meshes representing anatomical shape data. This application provides a flexible engine for viewing 3D triangulated meshes and calculating topographic metrics from individual files or directory batches. Shape is described via three metrics that characterize distinct aspects of form: curvature (Dirichlet Normal Energy: Bunn et al., 2011); relief (Relief Index: Ungar and M’Kirera, 2003; Boyer, 2008); and complexity (Orientation Patch Count Rotated: Evans et al., 2007). Details on relevant methods can be found in the listed publications. To run the compiled executable version MorphoTester in Windows, unzip the MorphoTester directory to a desire location and run Morpho.exe. In OSX, run the supplied application bundle (this may be placed in the Applications folder for easy continued access). To run MorphoTester from source code, execute Morpho.py as a script via the Python interpreter (via the command “python morpho.py”). Sample data and known values are provided for testing the application. This software is provided under a GPL V2.0 (or later) license, see below for details.

**File Type and Size**

MorphoTester accepts .ply Stanford PLY format surface mesh files. Triangulated surface mesh files (that is, surfaces comprised of multiple interconnected triangular polygons in three-dimensional space) can be generally described by the number of triangular polygons comprising each mesh. MorphoTester may be slow to load surface meshes consisting of >500,000 faces or more depending on computer speed, and DNE implicit fair mesh smoothing will be very slow at >20,000 triangles. Previously published work using this software has analyzed surface meshes simplified to 10,000 faces with another application. Examples of applications capable of this include Amira, Aviso, or the freeware application Meshlab. Future versions of MorphoTester may include mesh simplification built in.

# Processing Single Files

1. Load single file by selecting the Open File button, navigating to desired file, and selecting Open. The surface can be inspected using the 3D viewer on the right.
2. Choose which topographic metrics are to be measured. Set parameters and optional procedures for DNE and OPCR calculation using the nearby Options button (See “DNE Options” and “OPCR Options” below for more detail).
3. Select Process File and wait. Values will be output shortly.

# Batch Processing Multiple Files

1. All .ply meshes to be measured should be located in a single directory.
2. Select ‘Open Directory’ and navigate to desired directory for analysis. Select Open. No mesh will appear in the 3D viewer.
3. As described above, choose which topographic metrics are to be measured, and use the Option menus to set parameters.
4. Select Process Directory, and wait. Values will be output shortly. If an error occurs, this process will halt entirely.

Batch processing produces a results file in the directory where analyzed files are located. Results are provided as a tab-delineated table of topographic values and file names, and may be opened in Microsoft Excel or other applications.

# DNE Options

If optional model smoothing for DNE is desired, check ‘DNE Implicit Fairing Smooth.’ All previously published DNE calculations employ this smoothing, with a smoothing iteration number of 3 and a step size of 0.1. This smoothing step can introduce possible application errors, but it can also help reduce surface mesh noise which can disproportionately affect DNE values. DNE can be calculated regardless of whether implicit fairing is enabled. If implicit fairing is not enabled, the iteration number and step size values are ignored.

Overall DNE can be disproportionately affected by intersections between polygons with extreme angles such as often results from mesh noise or erroneous sharp features on surface casts pre-scanning (see ‘Absurdly High DNE Values’ below). To address this, the ‘0.1% Outlier Removal’ option culls all individual DNE per polygonal face values above the 99.9^th^ percentile. Similarly, the ‘Condition number checking’ option removes individual DNE per polygonal face values when the matrix comprising the face has a high condition number. High conditions numbers can indicate a matrix is singular (meaning further calculation of DNE cannot continue) and/or that the particular polygonal intersection is unreliable as a shape indicator due to extreme changes in DNE as a result from minor changes in polygon position. ‘0.1% Outlier Removal’ can be toggled on and off as desired. Current publications of DNE do not use this feature, though future publications will. ‘Condition number checking’ should generally be left on, unless specific reasons indicate turning it off.

If Visualize DNE is checked, energy quantity values will be visualized across a mesh surface as a heatmap. Relative DNE visualization uses minimum and maximimum surface polygon energy quantities to bound the heatmap legend, and is useful to plotting DNE across an individual surface. Absolute DNE visualization allows the user to specify the bounds of the heatmap plotting and is useful for comparing DNE between two surfaces.

# OPCR Options

‘Minimum Patch Count’ defines the smallest size of a patch (in terms of number of triangles comprising the patch) that will be counted for OPC calculation (see Evans et al., 2007 for more detail). 3 is the default value. If ‘Visualize OPCR’ is checked, OPCR results for single files will be depicted as colored patches on the mesh surface in the 3D window viewer pane on the right. Patches are colored according to their aspect, with each of the eight colors representing an arc of 45 degrees. This visualization is similar to that provided by Evans et al. (2007), but is different in that it represents aspect-designated patches on a 3D triangular polygon mesh instead of a GIS grid of single Z escalation values associated with XY coordinate pairs.

**Changes from beta versions**

*Different RFI results between beta and current versions of MorphoTester:*

Compared to latest release versions, some older betas of MorphoTester generate different values for “outline area.” Outline area is the 2D area of a 3D surface as projected onto the XY plane (for dental analyses this is often the occlusal plane). MorphoTester calculates 2D projected area by producing a flat pixelated image of a surface, counting surface pixels, and then multiplying this count by an area to pixel ratio. The pixel counting method used here was updated midway through beta development to ensure compatibility between Windows and OSX environments. Any differences in outline area should be small, usually around 1%.

*Different DNE results with outlier removal between beta and current versions of MorphoTester:*

Unlike for RFI, the latest version of MorphoTester should be able to replicate all DNE results obtained from any beta version. The base DNE method is identical across all versions, but certain beta versions do use different protocols for removing outliers, polygons with extremely high energy values. Older beta versions removed polygons with energy values above the 99^th^ percentile across a surface mesh (for a mesh of 10,000 polygons, 100 outliers removed). Later beta versions changed this to only remove polygons with energy values above the 99.9^th^ percentile (for a mesh of 10,000 polygons, 10 outliers removed). In both of these cases, outliers were removed from values calculated as energy density multiplied by polygon face area. One beta version, 0.2.0d, removed outliers from values calculated as raw energy densities. The latest version of MorphoTester allows users to specify outlier percentile and whether outliers should be removed from energy densities * polygon areas or raw energy densities. Outlier removal at 99.9% using energy densities * polygon areas is currently recommended, but trends of differences between specimens should be generally similar regardless of approach.

# Known Issues

# *CHOL errors*

This is the primary bug likely to be encountered with MorphoTester. It will only be encountered when measuring DNE with implicit fairing smoothing. This error relates to the matrices that comprise the surface data, and in practice it has mostly been encountered as a result of smoothing operations completed by Amira or Aviso. The simplest solution to this problem, if implicit fairing is desired (for comparability to previous DNE results for example), is to not use smoothing functions from Amira or Aviso. Meshlab works equally well for this purpose. For models already encountering this error, applying a 1 or 2-iteration Laplacian smooth using Meshlab will fix the problem while not effecting DNE values significantly.

# *Absurdly high DNE values*

DNE can be sensitive to certain kinds of surface noise or mesh artifacts that are not biological, such as long thin gaps in surface models, triangular polygons overlapping one another or sitting at bizarre angles, or accessory isolated polygon regions distinct from the surface to be analyzed. This is not an issue with MorphoTester, but instead requires care in preparing surface meshes to reduce noise or remove non-biological surface errors. In previously published DNE results, 100 iterations of smoothing has been used on simplified 10,000 face polygonal models for this purpose.

# *Fullscreen crashes (OSX only)*

Application is known to crash sometimes on exiting full-screen visualization of 3D meshes.

*Errors in OPCR visualization (When interpreting source code only)*

If MorphoTester is run with the latest versions of Mayavi (4.4.3) and VTK (6) dependent packages, visualization of OPCR may generate error messages. This is due to a bug in the dependent packages that will hopefully be fixed in future versions. For the moment, it is effective to use an older version of Mayavi such as 4.3.

**License**

GNU GENERAL PUBLIC LICENSE

Version 2, June 1991

Copyright (C) 1989, 1991 Free Software Foundation, Inc., <http://fsf.org/>

51 Franklin Street, Fifth Floor, Boston, MA 02110-1301 USA

Everyone is permitted to copy and distribute verbatim copies

of this license document, but changing it is not allowed.

Preamble

The licenses for most software are designed to take away your

freedom to share and change it. By contrast, the GNU General Public

License is intended to guarantee your freedom to share and change free

software--to make sure the software is free for all its users. This

General Public License applies to most of the Free Software

Foundation's software and to any other program whose authors commit to

using it. (Some other Free Software Foundation software is covered by

the GNU Lesser General Public License instead.) You can apply it to

your programs, too.

When we speak of free software, we are referring to freedom, not

price. Our General Public Licenses are designed to make sure that you

have the freedom to distribute copies of free software (and charge for

this service if you wish), that you receive source code or can get it

if you want it, that you can change the software or use pieces of it

in new free programs; and that you know you can do these things.

To protect your rights, we need to make restrictions that forbid

anyone to deny you these rights or to ask you to surrender the rights.

These restrictions translate to certain responsibilities for you if you

distribute copies of the software, or if you modify it.

For example, if you distribute copies of such a program, whether

gratis or for a fee, you must give the recipients all the rights that

you have. You must make sure that they, too, receive or can get the

source code. And you must show them these terms so they know their

rights.

We protect your rights with two steps: (1) copyright the software, and

(2) offer you this license which gives you legal permission to copy,

distribute and/or modify the software.

Also, for each author's protection and ours, we want to make certain

that everyone understands that there is no warranty for this free

software. If the software is modified by someone else and passed on, we

want its recipients to know that what they have is not the original, so

that any problems introduced by others will not reflect on the original

authors' reputations.

Finally, any free program is threatened constantly by software

patents. We wish to avoid the danger that redistributors of a free

program will individually obtain patent licenses, in effect making the

program proprietary. To prevent this, we have made it clear that any

patent must be licensed for everyone's free use or not licensed at all.

The precise terms and conditions for copying, distribution and

modification follow.

GNU GENERAL PUBLIC LICENSE

TERMS AND CONDITIONS FOR COPYING, DISTRIBUTION AND MODIFICATION

0. This License applies to any program or other work which contains

a notice placed by the copyright holder saying it may be distributed

under the terms of this General Public License. The "Program", below,

refers to any such program or work, and a "work based on the Program"

means either the Program or any derivative work under copyright law:

that is to say, a work containing the Program or a portion of it,

either verbatim or with modifications and/or translated into another

language. (Hereinafter, translation is included without limitation in

the term "modification".) Each licensee is addressed as "you".

Activities other than copying, distribution and modification are not

covered by this License; they are outside its scope. The act of

running the Program is not restricted, and the output from the Program

is covered only if its contents constitute a work based on the

Program (independent of having been made by running the Program).

Whether that is true depends on what the Program does.

1. You may copy and distribute verbatim copies of the Program's

source code as you receive it, in any medium, provided that you

conspicuously and appropriately publish on each copy an appropriate

copyright notice and disclaimer of warranty; keep intact all the

notices that refer to this License and to the absence of any warranty;

and give any other recipients of the Program a copy of this License

along with the Program.

You may charge a fee for the physical act of transferring a copy, and

you may at your option offer warranty protection in exchange for a fee.

2. You may modify your copy or copies of the Program or any portion

of it, thus forming a work based on the Program, and copy and

distribute such modifications or work under the terms of Section 1

above, provided that you also meet all of these conditions:

a) You must cause the modified files to carry prominent notices

stating that you changed the files and the date of any change.

b) You must cause any work that you distribute or publish, that in

whole or in part contains or is derived from the Program or any

part thereof, to be licensed as a whole at no charge to all third

parties under the terms of this License.

c) If the modified program normally reads commands interactively

when run, you must cause it, when started running for such

interactive use in the most ordinary way, to print or display an

announcement including an appropriate copyright notice and a

notice that there is no warranty (or else, saying that you provide

a warranty) and that users may redistribute the program under

these conditions, and telling the user how to view a copy of this

License. (Exception: if the Program itself is interactive but

does not normally print such an announcement, your work based on

the Program is not required to print an announcement.)

These requirements apply to the modified work as a whole. If

identifiable sections of that work are not derived from the Program,

and can be reasonably considered independent and separate works in

themselves, then this License, and its terms, do not apply to those

sections when you distribute them as separate works. But when you

distribute the same sections as part of a whole which is a work based

on the Program, the distribution of the whole must be on the terms of

this License, whose permissions for other licensees extend to the

entire whole, and thus to each and every part regardless of who wrote it.

Thus, it is not the intent of this section to claim rights or contest

your rights to work written entirely by you; rather, the intent is to

exercise the right to control the distribution of derivative or

collective works based on the Program.

In addition, mere aggregation of another work not based on the Program

with the Program (or with a work based on the Program) on a volume of

a storage or distribution medium does not bring the other work under

the scope of this License.

3. You may copy and distribute the Program (or a work based on it,

under Section 2) in object code or executable form under the terms of

Sections 1 and 2 above provided that you also do one of the following:

a) Accompany it with the complete corresponding machine-readable

source code, which must be distributed under the terms of Sections

1 and 2 above on a medium customarily used for software interchange; or,

b) Accompany it with a written offer, valid for at least three

years, to give any third party, for a charge no more than your

cost of physically performing source distribution, a complete

machine-readable copy of the corresponding source code, to be

distributed under the terms of Sections 1 and 2 above on a medium

customarily used for software interchange; or,

c) Accompany it with the information you received as to the offer

to distribute corresponding source code. (This alternative is

allowed only for noncommercial distribution and only if you

received the program in object code or executable form with such

an offer, in accord with Subsection b above.)

The source code for a work means the preferred form of the work for

making modifications to it. For an executable work, complete source

code means all the source code for all modules it contains, plus any

associated interface definition files, plus the scripts used to

control compilation and installation of the executable. However, as a

special exception, the source code distributed need not include

anything that is normally distributed (in either source or binary

form) with the major components (compiler, kernel, and so on) of the

operating system on which the executable runs, unless that component

itself accompanies the executable.

If distribution of executable or object code is made by offering

access to copy from a designated place, then offering equivalent

access to copy the source code from the same place counts as

distribution of the source code, even though third parties are not

compelled to copy the source along with the object code.

4. You may not copy, modify, sublicense, or distribute the Program

except as expressly provided under this License. Any attempt

otherwise to copy, modify, sublicense or distribute the Program is

void, and will automatically terminate your rights under this License.

However, parties who have received copies, or rights, from you under

this License will not have their licenses terminated so long as such

parties remain in full compliance.

5. You are not required to accept this License, since you have not

signed it. However, nothing else grants you permission to modify or

distribute the Program or its derivative works. These actions are

prohibited by law if you do not accept this License. Therefore, by

modifying or distributing the Program (or any work based on the

Program), you indicate your acceptance of this License to do so, and

all its terms and conditions for copying, distributing or modifying

the Program or works based on it.

6. Each time you redistribute the Program (or any work based on the

Program), the recipient automatically receives a license from the

original licensor to copy, distribute or modify the Program subject to

these terms and conditions. You may not impose any further

restrictions on the recipients' exercise of the rights granted herein.

You are not responsible for enforcing compliance by third parties to

this License.

7. If, as a consequence of a court judgment or allegation of patent

infringement or for any other reason (not limited to patent issues),

conditions are imposed on you (whether by court order, agreement or

otherwise) that contradict the conditions of this License, they do not

excuse you from the conditions of this License. If you cannot

distribute so as to satisfy simultaneously your obligations under this

License and any other pertinent obligations, then as a consequence you

may not distribute the Program at all. For example, if a patent

license would not permit royalty-free redistribution of the Program by

all those who receive copies directly or indirectly through you, then

the only way you could satisfy both it and this License would be to

refrain entirely from distribution of the Program.

If any portion of this section is held invalid or unenforceable under

any particular circumstance, the balance of the section is intended to

apply and the section as a whole is intended to apply in other

circumstances.

It is not the purpose of this section to induce you to infringe any

patents or other property right claims or to contest validity of any

such claims; this section has the sole purpose of protecting the

integrity of the free software distribution system, which is

implemented by public license practices. Many people have made

generous contributions to the wide range of software distributed

through that system in reliance on consistent application of that

system; it is up to the author/donor to decide if he or she is willing

to distribute software through any other system and a licensee cannot

impose that choice.

This section is intended to make thoroughly clear what is believed to

be a consequence of the rest of this License.

8. If the distribution and/or use of the Program is restricted in

certain countries either by patents or by copyrighted interfaces, the

original copyright holder who places the Program under this License

may add an explicit geographical distribution limitation excluding

those countries, so that distribution is permitted only in or among

countries not thus excluded. In such case, this License incorporates

the limitation as if written in the body of this License.

9. The Free Software Foundation may publish revised and/or new versions

of the General Public License from time to time. Such new versions will

be similar in spirit to the present version, but may differ in detail to

address new problems or concerns.

Each version is given a distinguishing version number. If the Program

specifies a version number of this License which applies to it and "any

later version", you have the option of following the terms and conditions

either of that version or of any later version published by the Free

Software Foundation. If the Program does not specify a version number of

this License, you may choose any version ever published by the Free Software

Foundation.

10. If you wish to incorporate parts of the Program into other free

programs whose distribution conditions are different, write to the author

to ask for permission. For software which is copyrighted by the Free

Software Foundation, write to the Free Software Foundation; we sometimes

make exceptions for this. Our decision will be guided by the two goals

of preserving the free status of all derivatives of our free software and

of promoting the sharing and reuse of software generally.

NO WARRANTY

11. BECAUSE THE PROGRAM IS LICENSED FREE OF CHARGE, THERE IS NO WARRANTY FOR THE PROGRAM, TO THE EXTENT PERMITTED BY APPLICABLE LAW. EXCEPT WHEN OTHERWISE STATED IN WRITING THE COPYRIGHT HOLDERS AND/OR OTHER PARTIES PROVIDE THE PROGRAM "AS IS" WITHOUT WARRANTY OF ANY KIND, EITHER EXPRESSED OR IMPLIED, INCLUDING, BUT NOT LIMITED TO, THE IMPLIED WARRANTIES OF MERCHANTABILITY AND FITNESS FOR A PARTICULAR PURPOSE. THE ENTIRE RISK AS TO THE QUALITY AND PERFORMANCE OF THE PROGRAM IS WITH YOU. SHOULD THE PROGRAM PROVE DEFECTIVE, YOU ASSUME THE COST OF ALL NECESSARY SERVICING, REPAIR OR CORRECTION.

12. IN NO EVENT UNLESS REQUIRED BY APPLICABLE LAW OR AGREED TO IN WRITING WILL ANY COPYRIGHT HOLDER, OR ANY OTHER PARTY WHO MAY MODIFY AND/OR REDISTRIBUTE THE PROGRAM AS PERMITTED ABOVE, BE LIABLE TO YOU FOR DAMAGES, INCLUDING ANY GENERAL, SPECIAL, INCIDENTAL OR CONSEQUENTIAL DAMAGES ARISING OUT OF THE USE OR INABILITY TO USE THE PROGRAM (INCLUDING BUT NOT LIMITED TO LOSS OF DATA OR DATA BEING RENDERED INACCURATE OR LOSSES SUSTAINED BY YOU OR THIRD PARTIES OR A FAILURE OF THE PROGRAM TO OPERATE WITH ANY OTHER PROGRAMS), EVEN IF SUCH HOLDER OR OTHER PARTY HAS BEEN ADVISED OF THE POSSIBILITY OF SUCH DAMAGES.
